# A Ruthenium(II) Polypyridyl Complex Disrupts Actin Cytoskeleton Assembly and Blocks Cytokinesis

**DOI:** 10.1002/ange.202117449

**Published:** 2022-05-09

**Authors:** Martin R. Gill, Paul J. Jarman, Vanessa Hearnden, Simon D Fairbanks, Marcella Bassetto, Hannes Maib, John Palmer, Kathryn R. Ayscough, Jim A. Thomas, Carl Smythe

**Affiliations:** ^1^ Department of Chemistry Faculty of Science and Engineering Swansea University UK; ^2^ Department of Biomedical Science University of Sheffield UK; ^3^ Department of Materials Science and Engineering University of Sheffield UK; ^4^ Department of Chemistry University of Sheffield UK

**Keywords:** Actin, Cytokinesis, Cytoskeleton, Polypyridyl Complexes, Ruthenium

## Abstract

The dinuclear Ru^II^ complex [(Ru(phen)_2_)_2_(tpphz)]^4+^ (phen=1,10‐phenanthroline, tpphz=tetrapyridophenazine) “RuRuPhen” blocks the transformation of G‐actin monomers to F‐actin filaments with no disassembly of pre‐formed F‐actin. Molecular docking studies indicate multiple RuRuPhen molecules bind to the surface of G‐actin but not the binding pockets of established actin polymerisation inhibitors. In cells, addition of RuRuPhen causes rapid disruption to actin stress fibre organisation, compromising actomyosin contractility and cell motility; due to this effect RuRuPhen interferes with late‐stage cytokinesis. Immunofluorescent microscopy reveals that RuRuPhen causes cytokinetic abscission failure by interfering with endosomal sorting complexes required for transport (ESCRT) complex recruitment.

## Graphical abstract

The dinuclear Ru^II^ complex [(Ru(phen)_2_)_2_(tpphz)]^4+^ binds to G‐actin monomers, preventing assembly into F‐actin. This inhibits actin fibre assemblies within live cells and causes disruption to late cytokinesis by interfering with the function of endosomal sorting complexes required for transport (ESCRT) during abscission. These results reveal new possibilities for metal complexes as biomedical tools and novel therapeutic leads.

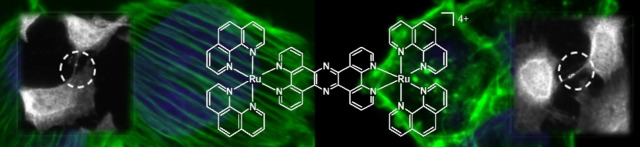

Actin dynamics are key to a wide range of cellular processes.[^1^, ^2^, ^3^, ^4^] Actin polymerisation inhibitors are vital in elucidating these processes and are potential therapeutic leads.[^5^, ^6^, ^7^, ^8^, ^9^] Examples include latrunculins, which bind to G‐actin monomers,[^10^, ^11^] and cytochalasins, which bind the end of actin filaments.^[12]^ The role of the actin cytoskeleton in cytokinesis—the final stage of cell division—is particularly important because cytokinesis errors produce aneuploidy or polyploidy; which can lead to cancer.^[13]^ Identification of inhibitors of these processes are actively sought and cytokinesis inhibitors are also being investigated as therapeutic leads.[^14^, ^15^] Although chemical screens have identified inhibitors of *early* cytokinesis,[^16^, ^17^] probes for *late* cytokinesis/abscission are still lacking. Consequently, this process remains one of the least well‐characterised within the cell‐cycle.[^18^, ^19^]

There is growing interest in the use of coordination compounds within chemical biology.[^20^, ^21^, ^22^] For example, ruthenium(II) polypyridyl complexes (RPCs) display a plethora of biomolecule targeting and intracellular functions.[^23^, ^24^, ^25^, ^26^, ^27^] The potential for RPCs targeting the cytoskeleton was first demonstrated by the MacDonnell group, which showed [Ru(DIP)_3_]^2+^ (DIP=4,7‐diphenyl‐1,10‐phenanthroline) functions as a microtubule stabilising agent, inhibiting microtubule assembly in live cells.^[28]^ In other work, Graminha et al. reported that a Ru^II^ gallic acid appeared to damage the actin cytoskeleton but the aetiology of this effect was not determined.^[29]^

Previous work has established [(Ru(phen)_2_)_2_(tpphz)]^4+^ (phen=1,10 phenanthroline, tpphz=tetrapyridophenazine) “RuRuPhen” (Figure 1) is internalised by live cells and images DNA.^[30]^ Interestingly, this treatment resulted in distinctive changes in cell morphology indicative of aberrations in cytoskeletal function. First, the effects of RuRuPhen on actin polymerisation in cell‐free conditions were examined using pyrene‐labelled G‐actin (Figure S1). Emission changes of this monomer can be used to monitor polymerisation to F‐actin.^[31]^ Addition of RuRuPhen resulted in a concentration‐dependent decrease in polymerisation rates and an increase in the lag time required for nucleation (Figures 2a, b). Due to the potential for energy transfer quenching of the pyrene excited state by RuRuphen, changes in the relative proportions of G‐ and F‐actin were also investigated by high speed centrifugation and protein electrophoresis.^[31]^ Strikingly, although RuRuPhen prevented the formation of F‐actin filaments from G‐actin (Figure 2c), addition of RuRuPhen to pre‐formed F‐actin resulted in no significant disassembly (Figure 2d), indicating that RuRuPhen inhibits G‐actin polymerisation without severing F‐actin.

**Figure 1:**
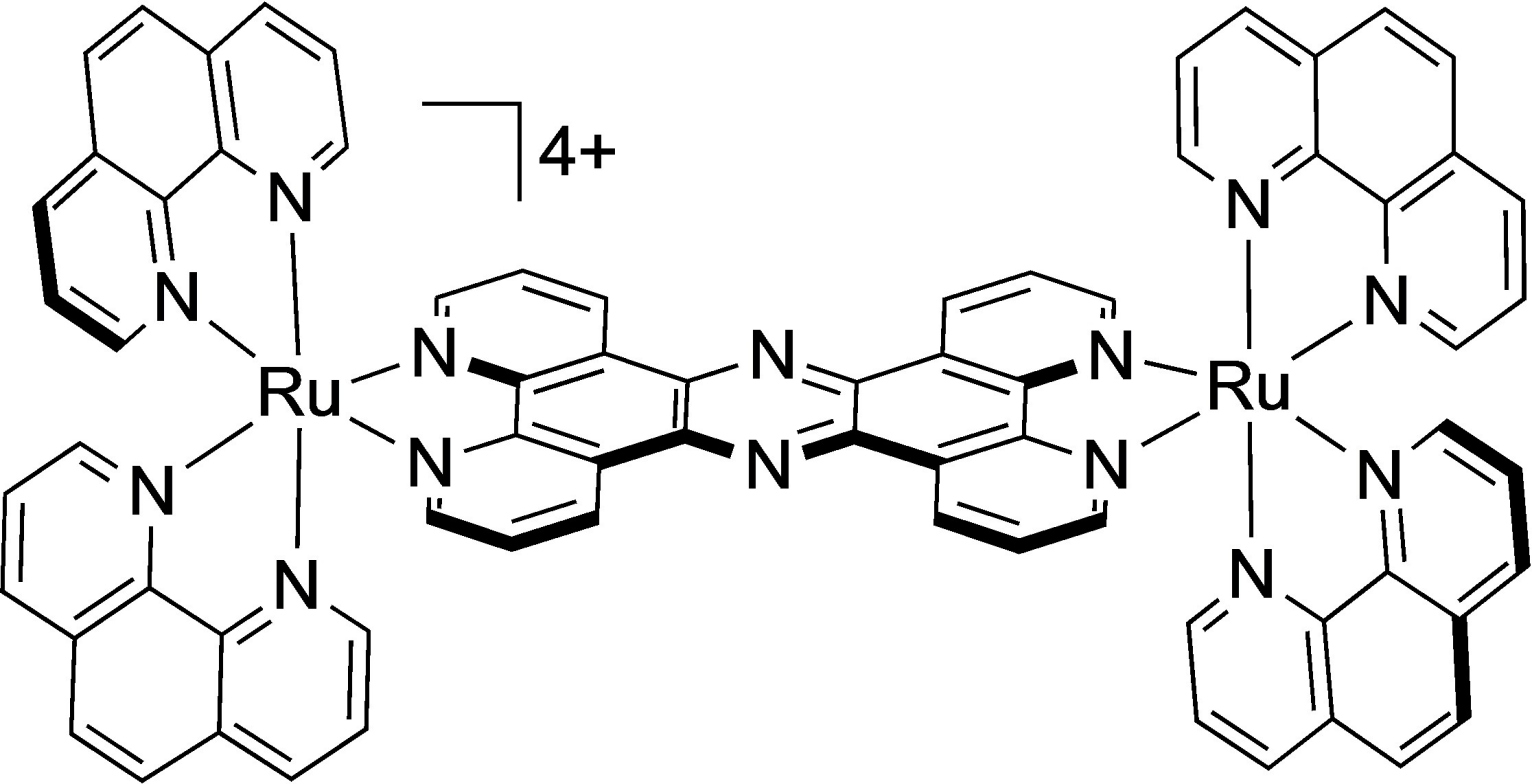
Structure of RuRuPhen. RuRuPhen was used as a mixture of stereoisomers in this work.

**Figure 2:**
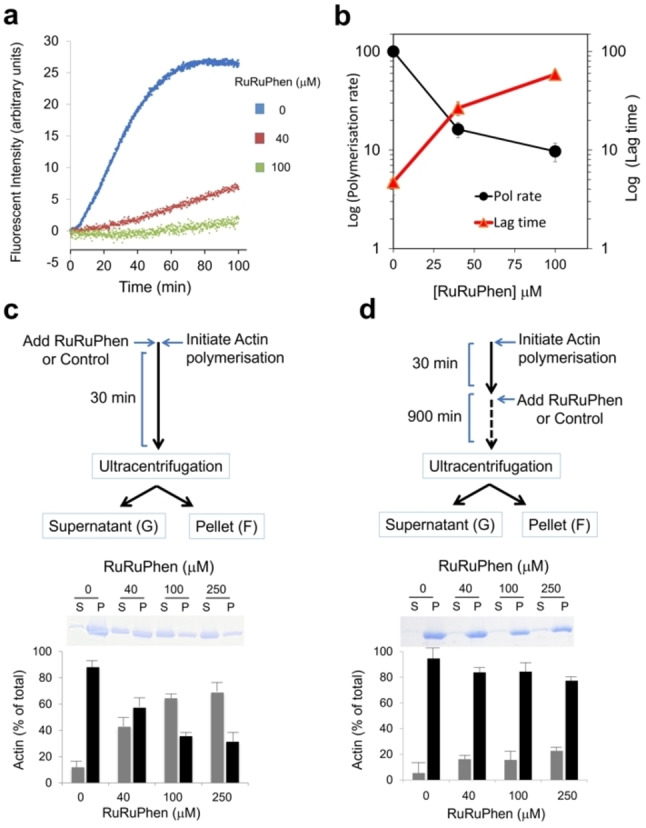
a) Pyrene‐actin polymerisation assay on addition of RuRuphen. b) Polymerisation rate and lag time as a function of RuRuPhen concentration. c) F‐actin formation determined by ultracentrifugation. Polymerisation was initiated by addition of KME buffer in the presence of RuRuPhen (RuRuPhen:actin ratios of 8 : 1, 20 : 1 and 50 : 1). After 30 min, samples were ultracentrifuged to separate F‐Actin (pellet) and G‐actin (supernatant). Protein content in each fraction was determined by Bradford assay (lower graph) in addition to SDS‐PAGE and Coomassie blue staining (upper panel). d) Addition of RuRuPhen to pre‐formed F‐actin, added after 30 min to samples at indicated concentrations and incubated for 900 min prior to F‐ and G‐ actin separation and quantitation as described in (c). Data mean +/− SD of three independent experiments.

In silico molecular docking studies were used to investigate RuRuPhen binding to G‐actin. No poses could be obtained within binding pockets of known substrates that sequester G‐actin and inhibit polymerisation (Latrunculin A, pectenotoxin 2 and reidispongiolide A), indicating that the rigid RuRuPhen is too large for any of the pockets (Figure S2). Therefore, blind docking was performed using the Dock tool of the molecular modelling MOE 2019.10 software,^[32]^ and the actin monomer structure PDB ID 2A42.^[33]^ Figures 3a, b show best scored poses obtained on the G‐actin surface. These multiple RuRuPhen binding sites include regions located on the edge of subdomains 4 and 3 and areas of contact between assembled subunits of F‐actin (Figure 3c),^[34]^ indicating that extensive RuRuPhen binding to G‐actin surfaces inhibits subunit interactions during polymerisation. This also explains the relatively high RuRuPhen: actin ratios required for cell‐free polymerisation inhibition.

**Figure 3:**
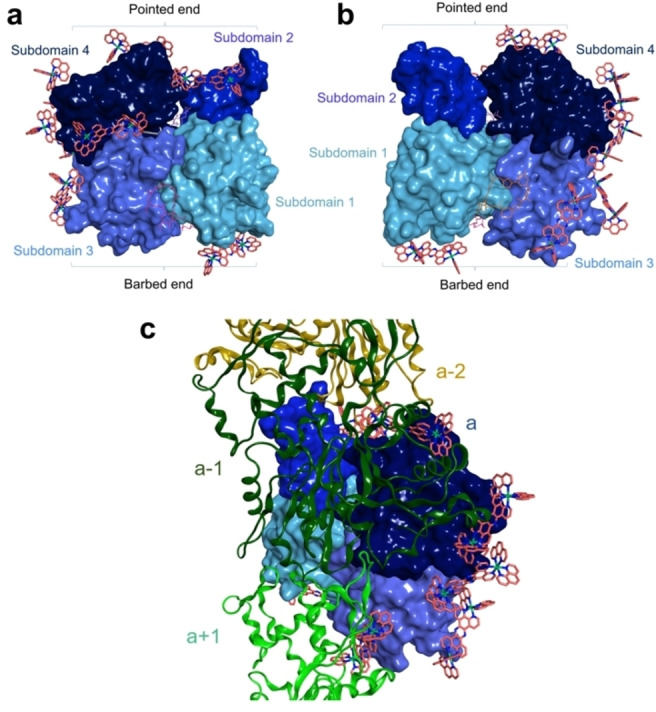
a), b) Eight best poses obtained in blind docking study using MOE 2019.10 and the 2 A42 crystal structure of actin monomer, front (a) and back (b). c) Superimposition of actin monomer shown in (a) and (b) with the F‐actin polymer structure (PDB ID 6DJN). Actin subunits are represented as coloured ribbon: green (a+1, barbed end), dark green (a‐1, pointed end), ochre (a‐2, pointed end).

Live cell uptake of RuRuPhen in a range of cell lines has already been established.[^30^, ^35^, ^36^] Inductively coupled plasma mass spectrometry analysis of treated A2780‐CP70 (referred to hereafter as CP70) cells revealed surprisingly high intracellular accumulation of RuRuPhen; within 10 mins, cells treated with 100 μM RuRuPhen display intracellular concentrations of >300 μM and concentrations approaching 950 μM are observed after 1 h exposure (Figure S3). After 1 h, RuRuPhen treatment (100 μM) resulted in substantial disruption of the actin cytoskeleton and the majority of treated cells lacked stress fibres (Figure 4a and Figure S4). Tubulin cytoskeletal structure in treated cells remained comparable to controls (Figure S5), indicating that the actin cytoskeleton is the preferential target of RuRuPhen. This effect was further examined using live cells transfected with the F‐actin probe LifeAct mRuby.^[37]^ HeLa cells were employed in these studies as CP70 cells could not be reliably transfected. Sites of cortical and stress fibre filaments were monitored though LifeAct mRuby fluorescence intensity. Initial addition of RuRuPhen produced a rapid (<30 s) transient increase in fluorescence in both locations (Figure 4b,c). Fluorescence from stress fibres then reduced rapidly, decreasing by ≈50 % within 300 s of treatment (Figure 4b, c).

**Figure 4:**
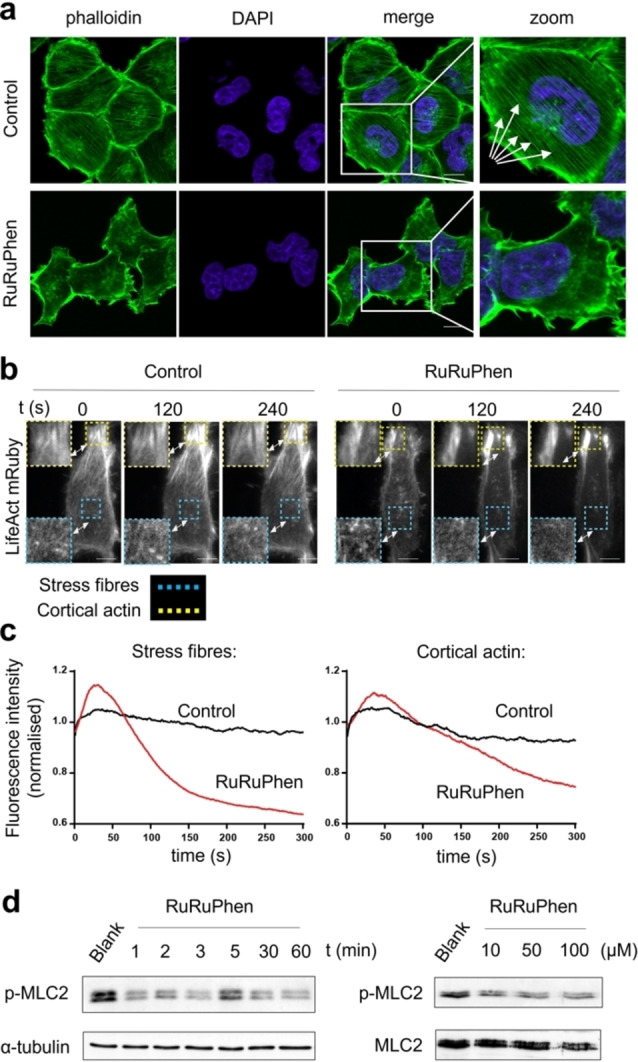
a) Actin cytoskeleton in RuRuPhen‐treated CP70 cells (100 μM, 1 h), determined by phalloidin staining and confocal microscopy. Right hand side: High‐magnification with arrows highlighting disruption to actin stress fibres. Scale bars=10 μm. b) Emission of HeLa cells transfected with F‐actin probe LifeAct mRuby measured following addition of RuRuPhen (500 μM) at selected time points. Scale bars=10 μm. c) Normalised fluorescence intensity at stress fibres (cyan boxes) and cortical actin (yellow boxes) at indicated times (0–300 s). d) Left, immunoblotting of whole cell extracts of CP70 cells treated with RuRuPhen (100 μM) at the stated incubation time for levels of activated p‐MLC2 (MLC2 phosphorylated at Ser19). α‐tubulin was used as a loading control. Right, p‐MLC2 (MLC2 phosphorylated at Thr18/Ser19) levels in cell lysates of cells treated with indicated concentrations of RuRuPhen (24 h). Levels of MLC independent of phosphorylation status are shown.

Phosphorylation of myosin light chain 2 (MLC2) plays a pivotal role in actin–myosin interactions and organisation of actin stress fibres.^[38]^ The impact of RuRuPhen on actomyosin contractility was examined by visualising levels of phosphorylated MLC2 in treated cells, which began to decrease after only one min of exposure (Figure 4d), a timing that corresponds to the disruption of actin stress fibre structure. These results show that RuRuPhen rapidly disrupts actin stress fibre organisation and actomyosin contractility and that the intracellular concentrations required for this bio‐activity are comparable to those in the cell‐free actin polymerisation inhibition studies.

Interestingly, time‐lapse videos of treated cells revealed defects in cytokinesis, the final stage of mitosis, with significant delays in abscission in which the midbody between two potential daughter cells remained intact for much longer than control cells (Figure 5a and Videos S1–S4). A 3.5‐fold increase in intact midbodies was quantified using indirect immunofluorescence microscopy (Figure 5b), and an intact midbody was even observed by transmission electron microscopy (Figure 5c). Importantly, no binuclear cells were observed, consistent with cells completing early stages of cytokinesis.^[17]^ This indicates that RuRuPhen interferes with *late* rather than *early* cytokinesis, thereby presenting a clear mechanistic distinction from previously reported cytokinesis inhibitors.[^39^, ^40^]

**Figure 5:**
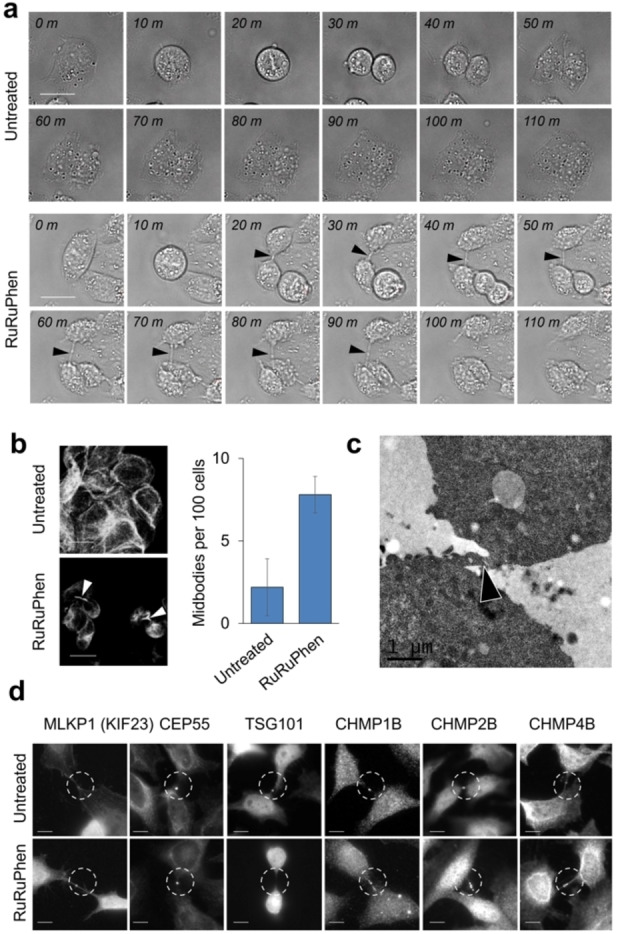
a) Time series of CP70 cells treated with control (upper panels) or 100 μM RuRuPhen (lower panels) showing visible midbody which remains intact for ≈70 mins before complete abscission. Scale bars=20 μm. b) Quantification of midbodies in RuRuPhen‐treated cells. After 16 h, CP70 cells were fixed, stained using anti α‐tubulin antibodies and the number of midbodies counted. Scale bars=20 μm. Average of duplicates, where at least 198 cells were counted per sample. c) TEM image of treated CP70 cell (100 μM, 24 h) showing intact midbody (arrow). d) Treated HeLa cells (100 μM, 10 h), fixed and stained with antibodies directed toward each of the indicated proteins involved in abscission. Images shown are representative of at least 30 cells examined. Scale bars=10 μm.

To identify the consequences of actin polymerisation inhibition on abscission machinery, we investigated recruitment of several cytokinesis‐related proteins by indirect immunofluorescence microscopy in HeLa cells, in which the molecular analysis of abscission has been best characterised.^[18]^ Recruitment of the central spindlin component MKLP1 and centrosome protein 55 (CEP55)—both essential for abscission[^41^, ^42^]—appeared normal in cells undergoing cytokinesis (Figure 5d). However, recruitment of the TSG101 component of the ESCRT‐I complex (ESCRT = endosomal sorting complexes required for transport) was reduced or absent on treatment, and although the ESCRT‐III subunits CHMP2A and CHMP4B, together with CHMP1B, were recruited to the midbody they were highly disorganised and the symmetric rings typically observed during abscission were absent (Figure 5d). That RuRuPhen causes cytokinetic abscission failure by interfering with the ESCRT complex recruitment and deployment at the mid‐body offers a corollary to a very recent report revealing the role of ESCRT in cytokinesis.^[43]^

Although actin is not commonly regarded as a therapeutic target,^[7]^ G‐actin polymerisation inhibitors that target cytoskeletal organisation exhibit considerable anti‐proliferative and anti‐metastatic activity.[^44^, ^45^] Within this context, in addition to blocking cytokinesis, RuRuPhen also inhibits cell spreading and motility (Figure S6). Together, these effects impacted cell viability of three cancer cell lines with potencies comparable to cisplatin but with a significantly reduced effect on a non‐cancer cell line (Figure S7 and Table S1). To combat resistance mechanisms, anti‐proliferatives that do not involve apoptosis or genomic DNA damage are sought as therapeutics.[^46^, ^47^, ^48^, ^49^, ^50^] So, proliferation inhibition by RuRuPhen accompanied by an increase in the proportion of cells in G1 phase without apoptosis or DNA damage response signalling (Figures S8 and S9) is significant. Cell shape disruption associated with loss of cytoskeletal integrity can trigger a G1 arrest^[51]^ and abscission‐stalled cells appear as two cells with G1 content in flow cytometry. Our results indicate a dual‐mode mechanism of action, combining loss of actin cytoskeletal integrity with a late cytokinesis block. This latter observation is significant as cytokinesis inhibitors should avoid early blocking which can lead to the aneuploidy associated with tumorigenesis.^[52]^

We can report that the previously reported complex [(Ru(DMP)_2_)_2_(tpphz)]^4+^ (DMP=2,9‐dimethyl‐1,10 phenanthroline)^[53]^ has a comparable impact on CP70 cell morphology, actomyosin contractility and cell proliferation to RuRuPhen (Figures S7, S10, Table S1). However, one notable difference is that RuRuDMP generates a less severe disruption of actin stress fibres than observed for RuRuPhen (Figure S10b). Interestingly, MacDonnell and colleagues reported [Ru(DIP)_3_]^2+^ interferes with microtubule dynamics in vitro by stabilising microtubules, and in vivo by interfering with microtubule growth, similar to the behaviour of taxanes.^[28]^ Taken together, one intriguing possibility raised by the MacDonnell Group's work on [Ru(DIP)_3_]^2+^, our studies herein, and the ruthenium(II) gallic acid complex reported by Graminha et al.^[29]^ is that that metal complexes of this kind represent a hitherto unidentified class of broad‐spectrum function modulators for cytoskeletal proteins.

However, it does seem these effects are quite specific; for the complexes described herein, no real changes in microtubule morphology were evidenced. While RuRuPhen‐treated cells displayed slight alterations in interphase microtubules morphology (see Figure S5), these are more likely explained by indirect changes arising from the dramatic loss of actin stress fibres. Significantly, exposure to RuRuPhen does not generate phenotypes closely associated with tubulin polymerisation inhibition such as cells arrested in mitosis, binucleate cells or apoptotic remnants.^[54]^ So although we cannot currently rule out the possibility that RuRuPhen also disrupts microtubule dynamics, it is unlikely that its effect on cytokinesis is the consequence of modulation of tubulin function, although experiment to definitively establish this hypothesis are underway.

Finally, as is the usual custom, the work described here was carried out on a mixture of the stereoisomers of RuRuPhen. However—as we have established that the interaction of closely related dinuclear complexes with biomolecules is highly dependent on the stereochemistry of each individual metal centre[^55^, ^56^]—the effects of individual stereoisomers of RuRuPhen on cell morphology is currently being investigated. Such studies will form the basis of future reports.

## Conflict of interest

The authors declare no conflict of interest.

## Supporting information

As a service to our authors and readers, this journal provides supporting information supplied by the authors. Such materials are peer reviewed and may be re‐organized for online delivery, but are not copy‐edited or typeset. Technical support issues arising from supporting information (other than missing files) should be addressed to the authors.

Supporting Information

Supporting Information

Supporting Information

Supporting Information

Supporting Information

## Data Availability

The data that support the findings of this study are available from the corresponding author upon reasonable request.
